# Resolution of recurrent pancreatitis and nutritional recovery with CFTR modulator therapy in CFTR-related disorder: a case report

**DOI:** 10.3389/fped.2026.1815310

**Published:** 2026-07-22

**Authors:** Folashade A. Jose

**Affiliations:** Pediatric Gastroenterology, Hepatology & Nutrition, Levine Children’s Hospital, Charlotte, NC, United States

**Keywords:** case report, CFTR-related disorder, elexacaftor/tezacaftor/ivacaftor, non-classic cystic fibrosis, nutritional recovery, recurrent pancreatitis

## Abstract

**Introduction:**

Cystic fibrosis transmembrane conductance regulator (CFTR) modulators have transformed cystic fibrosis care, but their role in CFTR-related disorders is not well defined.

**Case presentation:**

This is a 19 year old man who presented with recurrent acute pancreatitis beginning at 12–14 years of age, with no history of sinusitis, pneumonia, or asthma. He had poor weight gain, indeterminate sweat chloride values (43–49 mmol/L), and carried a single F508del CFTR mutation. He experienced recurrent episodes of parotitis between 8 and 10 years of age. Despite preserved exocrine pancreatic function and the absence of pulmonary symptoms, he experienced persistent nutritional failure despite appetite stimulation (cyproheptadine and mirtazapine). At 18 years of age, elexacaftor/tezacaftor/ivacaftor (ETI) was initiated based on genotype eligibility and the patient's clinical phenotype. Over 19 months, he achieved complete resolution of pancreatitis and substantial weight gain (54.9–69.2 kg; BMI 19.1–23.7 kg/m^2^). Pulmonary function remained normal and sweat chloride decreased to 42 mmol/L.

**Conclusion:**

This case highlights the potential benefit of CFTR modulators in CFTR-related disorders and supports consideration of therapy in selected non-classic presentations. Although weight gain began after initiation of mirtazapine, it continued following ETI and was accompanied by improvement in gastrointestinal symptoms and resolution of pancreatitis episodes.

## Introduction

Cystic fibrosis transmembrane conductance regulator (CFTR)-related disorders encompass a spectrum of phenotypes associated with mutations in the CFTR gene, often presenting with non-pulmonary manifestations such as pancreatitis. Partial dysfunction of the CFTR protein leads to altered ion transport and organ-specific complications (1). Historically, management has been supportive, focusing on symptom control rather than addressing the underlying defect (2). The advent of highly effective CFTR modulators, including elexacaftor/tezacaftor/ivacaftor (ETI), has shifted treatment toward targeting the underlying defect itself (3, 4). While their benefits are well established in classic cystic fibrosis, the role of CFTR modulators in CFTR-related disorders, particularly pancreatic manifestations, remains an area of ongoing clinical interest.

## Case presentation

A 19-year-old male presented with recurrent acute pancreatitis beginning at 12–14 years, with no history of sinusitis, pneumonia, or asthma. He had parotitis episodes at 8–10 years.

Initial genetic evaluation included sequence analysis of PRSS1, SPINK1, CTRC, and CFTR genes. Testing identified a heterozygous pathogenic CFTR variant (p.Phe508del). No pathogenic variants were detected in PRSS1, SPINK1, or CTRC.

CFTR analysis confirmed carrier status for p.Phe508del; however, intronic or splice-modifying variants (including poly-T and TG repeat status) were not reported, so additional variants affecting CFTR expression could not be excluded.

Sweat chloride testing was subsequently performed in this context and was indeterminate on two occasions (43 and 49 mmol/L). In the absence of a second identified CFTR mutation or documented splice-modifying variants, the findings were most consistent with a CFTR-related disorder rather than classic cystic fibrosis.

Pulmonary function tests at age 16 were technically limited, but repeat testing 7 months later showed normal spirometry (FEV1 106% predicted). Gastrointestinal symptoms included nausea, early satiety, poor appetite, and intermittent weight loss, without diarrhea or steatorrhea. Weight remained ∼116 lb (4th percentile) at ages 16 and 18, height was at the 18.5th percentile, and BMI at the 3.46th percentile. Imaging was normal. Fecal elastase was consistently >200 μg/g, indicating preserved exocrine pancreatic function. Endoscopy was performed to evaluate persistent upper gastrointestinal symptoms and poor growth, revealing mild chronic gastritis and generalized disaccharidase deficiency. Colonoscopy was not performed, as there were no lower gastrointestinal symptoms to suggest colonic pathology.

Genetic counseling concluded that CFTR variant was unlikely to be causative of pancreatitis, given the presence of a single mutation, a negative pancreatitis panel, and the absence of classic cystic fibrosis features.

Management included pancreatic enzyme replacement therapy (PERT) and cyproheptadine. PERT was initiated empirically for abdominal symptoms and nutritional concerns, despite normal fecal elastase, although adherence was inconsistent. Weight gain remained limited. Mirtazapine was initiated for appetite/anxiety with intermittent improvements. In May 2023 (age 18), ETI was initiated based on indeterminate sweat chloride values and persistent nutritional failure (3). Following ETI therapy, appetite, energy, and GI symptoms markedly improved, with complete resolution of pancreatitis. Over 19 months, weight increased from 54.9 kg to 69.2 kg, and BMI rose from 19.1 to 23.7 kg/m^2^ (4). Spirometry improved (FEV1 from 104% to 113% predicted), sweat chloride decreased to 42 mmol/L. Although weight gain began after initiation of mirtazapine, it continued following ETI and was accompanied by improvement in gastrointestinal symptoms and resolution of pancreatitis episodes.

## Discussion

This case describes the clinical course following initiation of ETI in a patient with a CFTR-related disorder characterized by recurrent pancreatitis and poor weight gain in the absence of pulmonary disease. While a single CFTR mutation does not meet diagnostic criteria for cystic fibrosis, heterozygous variants have been associated with CFTR-related disorders, particularly pancreatitis.

In this patient, the initial genetic interpretation did not support a causal role for CFTR. This highlights the importance of considering genetic results alongside the overall clinical picture, rather than relying on genetic findings in isolation.

An important limitation is that intronic splice modifiers, such as poly-T and TG repeat variants, were not characterized. Because these variants can influence CFTR function, they may have contributed to the patient's phenotype, limiting our ability to fully interpret the genotype-phenotype relationship. In addition, the absence of pathogenic variants in pancreatitis-associated genes (PRSS1, SPINK1, CTRC) further supports consideration of CFTR dysfunction as a contributing factor in this patient's recurrent pancreatitis.

Notwithstanding this uncertainty, the patient's clinical features—including recurrent pancreatitis, growth failure, and indeterminate sweat chloride levels—raised concern for clinically meaningful CFTR dysfunction and supported consideration of therapy.

Although a single F508del variant is insufficient for a diagnosis of cystic fibrosis, heterozygous CFTR mutations have been associated with increased risk of pancreatitis. In this context, partial CFTR dysfunction may impair bicarbonate and fluid secretion within the pancreatic ducts, predisposing to protein plugging, ductal obstruction, and recurrent inflammation (1, 3, 5). This case underscores the importance of interpreting genetic findings within the broader clinical context, rather than relying on genetic results in isolation.

**Figure 1:**
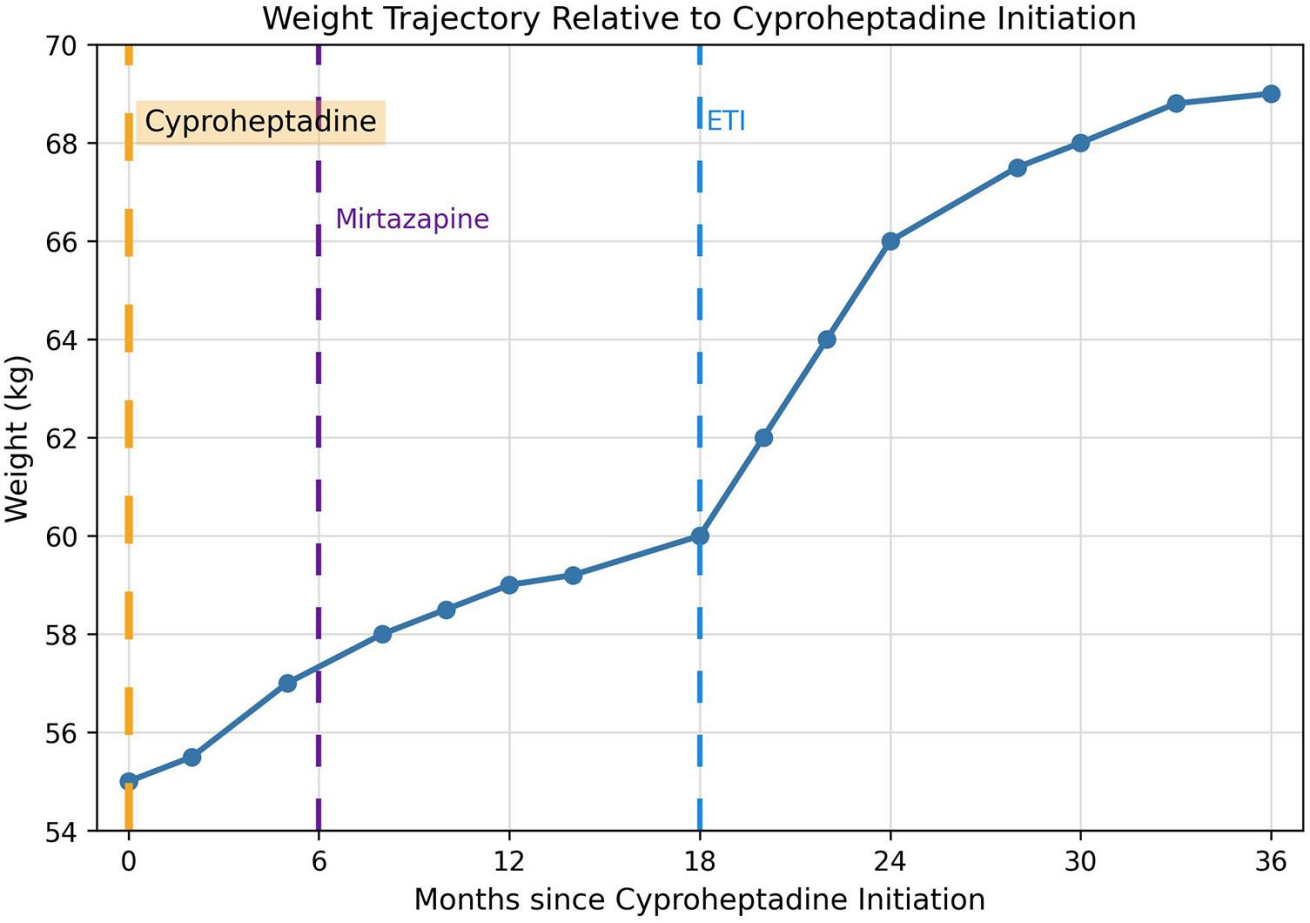
Longitudinal weight measurements from 2017 to 2024 with annotated timing of key interventions, including cyproheptadine (November 2021), mirtazapine (May 2022), and ETI initiation (May 2023). The trajectory demonstrates modest weight gain following mirtazapine and continued increase after ETI, in parallel with improvement in gastrointestinal symptoms and resolution of pancreatitis episodes.

Following ETI initiation (Table 1 and Figure 1), the patient experienced resolution of pancreatitis episodes and sustained improvement in nutritional status.

**Table 1:** Weight trajectory and intervention timeline showing approximate weight and BMI changes across treatment phases.

Phase	Timeframe	Intervention(s)	Weight (kg)	BMI (kg/m^2^)
Baseline	2,017–2,021	None	50–54	18.5–19.0
Cyproheptadine only	2,021–2,022	Cyproheptadine	54.0–54.9	19.0–19.1
Mirtazapine only	2,022–2,023	Mirtazapine	54.9–62.0	19.1–21.5
Post-ETI initiation	2,023–2,024	ETI + Mirtazapine	62.0–69.2	21.5–23.7

Key interventions were initiated at the following time points: cyproheptadine (November 2021), mirtazapine (May 2022), and elexacaftor/tezacaftor/ivacaftor (ETI; May 2023). More detailed longitudinal measurements are reflected in Figure 1.

Weight gain in this case should be interpreted cautiously. The patient experienced a similar degree of weight gain during treatment with mirtazapine prior to initiation of ETI, suggesting that appetite stimulation is an important potential contributor. However, the clinical course after starting ETI was notable for complete resolution of recurrent pancreatitis, which had not occurred during earlier treatment phases. The patient and family also described more sustained improvements in appetite, energy, and overall gastrointestinal symptoms following ETI. Weight gain during this period occurred alongside stability in pancreatic symptoms, raising the possibility of a broader therapeutic effect beyond appetite stimulation alone.

While mirtazapine likely contributed to early weight gain, the temporal association between ETI initiation, resolution of pancreatitis, and continued nutritional improvement suggests a possible contribution from CFTR modulation. As with any single case, causality cannot be established, and the relative contributions of appetite stimulation and disease modification are difficult to separate.

The relatively modest reduction in sweat chloride further suggests that this biomarker may not fully capture clinical response in CFTR-related disorders. In such cases, organ-specific outcomes—including pancreatic manifestations and nutritional status—may provide more clinically meaningful indicators of response.

Emerging studies support a broader role for CFTR modulators beyond classic cystic fibrosis, including improvement in CFTR function in experimental pancreatic models and reported benefit in patients with CFTR-variant-associated pancreatitis (5). Further prospective studies are needed to better define the role of CFTR modulators in patients with CFTR-related disorders presenting with pancreatic manifestations. Key clinical events and weight trajectory are summarized in Table 1 and displayed in detail in Figure 1.

## Conclusion

ETI therapy was associated with resolution of recurrent pancreatitis and significant nutritional recovery in a young adult with a CFTR-related disorder lacking classic pulmonary symptoms. This case supports consideration of CFTR modulator therapy in selected patients with CFTR-related pancreatitis and highlights the need for further study in this population.

## Patient perspective

The patient reported improved appetite, energy, and quality of life after starting ETI. He noted relief from recurrent abdominal pain and gained the ability to maintain weight during the transition to college.

## Data Availability

The raw data supporting the conclusions of this article will be made available by the authors, without undue reservation.
